# The Sinus Snare: A Case Series of Isolated Sphenoid Sinusitis Presenting as Orbital Apex Syndrome in Poorly Controlled Diabetes

**DOI:** 10.7759/cureus.101275

**Published:** 2026-01-11

**Authors:** Monica Jawahar, Rajasekar MK

**Affiliations:** 1 Otolaryngology-Head and Neck Surgery, Sree Balaji Medical College and Hospital, Chennai, IND

**Keywords:** diabetes type 2, orbital apex syndrome, paranasal sinusitis, sphenoid sinusitis, uncontrolled diabetes

## Abstract

Isolated sphenoid sinusitis is an uncommon entity, accounting for a small proportion of paranasal sinus disease. Its deep anatomical location and proximity to the optic nerve, cavernous sinus, and orbital apex often result in delayed diagnosis, particularly when typical nasal symptoms are absent. In poorly controlled diabetics, fungal infection of the sphenoid sinus can progress rapidly, presenting initially with ophthalmic deficits rather than sinonasal complaints. This series describes three female patients between 50 and 53 years of age, all with long-standing type 2 diabetes mellitus with irregular treatment compliance. Each patient presented primarily with visual disturbance: the first with gradual blurring of vision and mild proptosis; the second with acute-onset ptosis, proptosis, and diplopia indicative of orbital apex syndrome; and the third with isolated lateral gaze diplopia due to abducens nerve palsy. Nasal symptoms were minimal or absent in all three cases. Imaging demonstrated isolated sphenoid sinus involvement, and diagnostic nasal endoscopy revealed subtle posterior drainage signs. Endoscopic sphenoidotomy was performed in all patients. Histopathology confirmed mucormycosis in one patient and Aspergillus fungal sinusitis in another. Postoperative outcomes were favorable, with all three patients achieving improvement of visual acuity to 6/6 and complete resolution of ophthalmoplegia where present. This case series emphasizes that visual symptoms in diabetics may be the earliest manifestation of sphenoid sinus disease. Early radiologic evaluation and prompt endoscopic surgical intervention, coupled with appropriate antifungal therapy, are essential to prevent permanent optic nerve injury and ensure favorable neurological and visual outcomes.

## Introduction

The sphenoid sinus occupies a uniquely deep and centrally positioned anatomical location within the skull base, rendering it both structurally significant and clinically challenging to evaluate. Although it lies in close proximity to several vital neurovascular structures--including the optic nerve, cavernous sinus, internal carotid artery, and cranial nerves II, III, IV, V1, V2, and VI--pathology arising from the sphenoid sinus remains comparatively uncommon when contrasted with disease involving the maxillary, frontal, or anterior ethmoidal sinuses [1]. Isolated sphenoid sinusitis accounts for less than 3% of all paranasal sinus infections, yet the potential severity of its consequences is substantial. Even subtle disease within this confined space may lead to serious ophthalmic or neurological complications if early clinical signs are overlooked or misdiagnosed [1]. The deep location of the sphenoid sinus often results in delayed symptom recognition and difficulty with clinical and radiological evaluation, thereby allowing disease progression toward adjacent critical structures.

Infections confined to the sphenoid sinus demonstrate a heterogeneous and often deceptively nonspecific clinical presentation. Unlike typical paranasal sinusitis, classical sinonasal manifestations such as nasal obstruction, rhinorrhea, or postnasal drip are frequently absent or minimal [2]. Consequently, patients may present with vague complaints, most commonly persistent, poorly localized headaches that can be frontal, temporal, vertex, occipital, or retro-orbital in distribution [2]. This broad symptom overlap often leads clinicians to consider alternative diagnoses, including primary headache syndromes, trigeminal neuralgia, tension-type headaches, or intracranial lesions, thereby contributing to missed or delayed recognition of sphenoid sinus disease [3]. In many patients, ophthalmic manifestations, such as blurring of vision, diplopia, or cranial nerve palsies, constitute the earliest clear indicators of disease extension beyond the confines of the sinus, particularly when inflammation or infection involves the orbital apex or cavernous sinus [3].

Long-term institutional experience further underscores the under-recognition of isolated sphenoid sinusitis. Retrospective analyses reveal that many patients undergo repeated evaluations at multiple healthcare facilities before an accurate diagnosis is made, often receiving symptomatic treatment for unrelated disorders due to the ambiguous nature of early symptoms [4]. The rarity of the disease, combined with its subtle and variable presentation, contributes to the underestimation of its clinical significance. Without timely detection, sphenoid sinus infections may progress via direct bony erosion, venous pathways, or perineural spread to adjacent orbital or intracranial structures, resulting in permanent neurological or visual morbidity [3].

The spectrum of isolated sphenoid sinus pathology is broad, encompassing acute bacterial infections, fungal sinusitis, mucoceles, polyps, and neoplastic conditions. Among these, infectious etiologies predominate. Recognition of the specific causative organism is essential because outcomes differ markedly between bacterial sinusitis, non-invasive fungal sinus disease, and acute invasive fungal sinusitis. The latter has become increasingly important in contemporary clinical practice, particularly among immunocompromised individuals and patients with poorly controlled diabetes mellitus, in whom rapid tissue invasion, vascular thrombosis, and necrosis may occur. Differentiating invasive from non-invasive fungal pathology at the earliest stage is critical, as delays in surgical and antifungal therapy may lead to rapid deterioration and irreversible complications [5].

Given the sphenoid sinus's intimate proximity to structures vital for visual function, spread of infection from the posterior ethmoid or sphenoid sinus to the orbital apex, cavernous sinus, or optic nerve sheath can precipitate severe orbital complications. One of the most dramatic and vision-threatening consequences is orbital apex syndrome, characterized by the simultaneous involvement of the optic nerve and multiple extraocular motor nerves, resulting in visual loss accompanied by ophthalmoplegia [6]. Clinical manifestations may include decreased visual acuity, afferent pupillary defects, ptosis, external ophthalmoplegia, and sensory deficits in the ophthalmic division of the trigeminal nerve [6]. Because visual compromise may progress rapidly, prompt clinical evaluation and intervention are critical to preserving function.

Management of orbital apex lesions necessitates coordinated multidisciplinary care involving ophthalmology, otolaryngology, and neurology. Radiological evaluation with computed tomography and magnetic resonance imaging provides essential information regarding the extent of sinus involvement, presence of bony erosion, and orbital or cavernous sinus extension. Early surgical intervention, most commonly via functional endoscopic sinus surgery (FESS), plays a central role in decompressing the sphenoid sinus, restoring drainage, and removing infected or devitalized tissue [7]. Delayed treatment increases the likelihood of severe complications, including optic neuropathy, cavernous sinus thrombosis, meningitis, or intracranial abscess formation, all associated with significant morbidity and mortality [8].

The classification of fungal rhinosinusitis highlights the distinction between non-invasive forms (such as fungal balls or allergic fungal sinusitis) and invasive fungal disease, which may be acute, fulminant, or chronic in nature. Acute invasive fungal sinusitis, in particular, is strongly associated with immunosuppressive states, including poorly controlled diabetes, hematologic malignancies, transplant-related immunosuppression, and prolonged corticosteroid therapy. In this high-risk population, fungal organisms rapidly permeate tissue planes and vasculature, resulting in infarction, necrosis, and high mortality despite advances in surgical and medical therapy. Although survival may improve with prompt diagnosis and aggressive treatment, visual recovery is closely tied to the duration and severity of optic nerve compromise prior to intervention [9].

In India, the incidence of fungal rhinosinusitis continues to rise, especially among individuals with uncontrolled diabetes mellitus. Environmental fungal exposure, humid climatic conditions, and delays in seeking specialized care contribute to advanced-stage presentation. Many patients present only after developing alarming symptoms, such as visual loss or cranial nerve dysfunction, emphasizing the need for heightened clinical suspicion, particularly in diabetic individuals presenting with ocular complaints or persistent headaches lacking overt sinonasal symptoms.

This case series presents three diabetic patients with isolated sphenoid sinusitis who primarily manifested with ophthalmic symptoms, including blurring of vision, diplopia, and features of orbital apex involvement. All patients had a history of poor glycemic control, likely predisposing them to infectious sphenoid sinus pathology and subsequent orbital complications. This series aims to underscore the subtle yet serious nature of isolated sphenoid sinus disease, emphasize its potential for significant ophthalmic morbidity, and highlight the critical importance of early radiologic assessment and prompt surgical management in preventing irreversible visual impairment.

## Case presentation

This case series describes three middle-aged female patients with long-standing or newly diagnosed type 2 diabetes mellitus (T2DM) who presented with ophthalmic manifestations secondary to isolated sphenoid sinus disease. Their baseline characteristics and presenting symptoms are summarized in Table 1, and corresponding endoscopic and radiological findings are detailed in Table 2.

**Table 1:** Baseline patient characteristics and presenting symptoms of the cases. LE: left eye; OHA: oral hypoglycemic agents; PR: pulse rate; RA: room air; RE: right eye; T2DM: type 2 diabetes mellitus.

Parameter	Case 1	Case 2	Case 3
Age/sex	53/female	52/female	50/female
Diabetes status	Newly diagnosed T2DM; irregular OHA use	T2DM 12 years; irregular medication	T2DM 25 years; irregular medication
Other comorbidities	None reported	Systemic hypertension, bronchial asthma	None reported
Primary presenting complaint	Gradual blurring of vision (right eye) for four months	Acute right orbital swelling with blurring of vision and diplopia for one week	Diplopia and blurred vision (right eye) for two weeks
Headache	Present; dull frontal-occipital	Present; right-sided	Occasional; right-sided
Nasal symptoms	Minimal, intermittent nasal block	None significant	None significant
Fever/systemic symptoms	Absent	Absent	Absent
Vitals at presentation	BP 130/90 mmHg; PR 84 bpm; SpO₂ 98% RA	BP 130/90 mmHg; PR 84 bpm; SpO₂ 98% RA	BP 110/70 mmHg; PR 72 bpm; SpO₂ 98% RA
Visual acuity (pre-operative)	RE 6/12 → 6/9; LE 6/18 → 6/6	RE 6/18 → 6/9; LE 6/6	RE 6/18 → 6/9; LE 6/6
Proptosis	Mild, right	Marked, right	Not present
Diplopia	Absent	Present	Present (on lateral gaze)
Cranial nerve involvement	None	III, IV, VI (orbital apex syndrome)	VI palsy (right)

**Table 2:** Endoscopic findings and radiological evaluation of the cases. DNS: deviated nasal septum; MT: middle turbinate; PNS: paranasal sinuses.

Parameter	Case 1	Case 2	Case 3
Diagnostic nasal endoscopy	Mucopurulent discharge from right sphenoethmoidal recess; DNS to left with spur	High right septal deviation; polypoidal right MT; mucoid discharge in right middle meatus	High septal deviation (right > left); minimal mucosal congestion
CT PNS findings	Isolated right sphenoid sinus opacification; no bony erosions	Sphenoid sinus disease with extension toward orbital apex	Isolated right sphenoid sinus opacification
MRI findings	Not documented	MRI brain with contrast showing orbital involvement; no cavernous sinus thrombosis recorded	Not done/not documented
Orbit/neurological extension	None	Present (orbital apex involvement)	None
Intracranial complications	None observed	None documented	None documented

Case 1

A 53-year-old woman with recently diagnosed type 2 diabetes mellitus, poorly controlled on irregular oral hypoglycemic therapy, presented with gradually progressive blurring of vision in the right eye over four months. This visual deterioration was accompanied by right-sided facial heaviness and a dull frontal-occipital headache. The absence of nasal obstruction, rhinorrhea, fever, or otological symptoms was notable and is consistent with typical presentations of posterior sinus disease.

**Figure 1:**
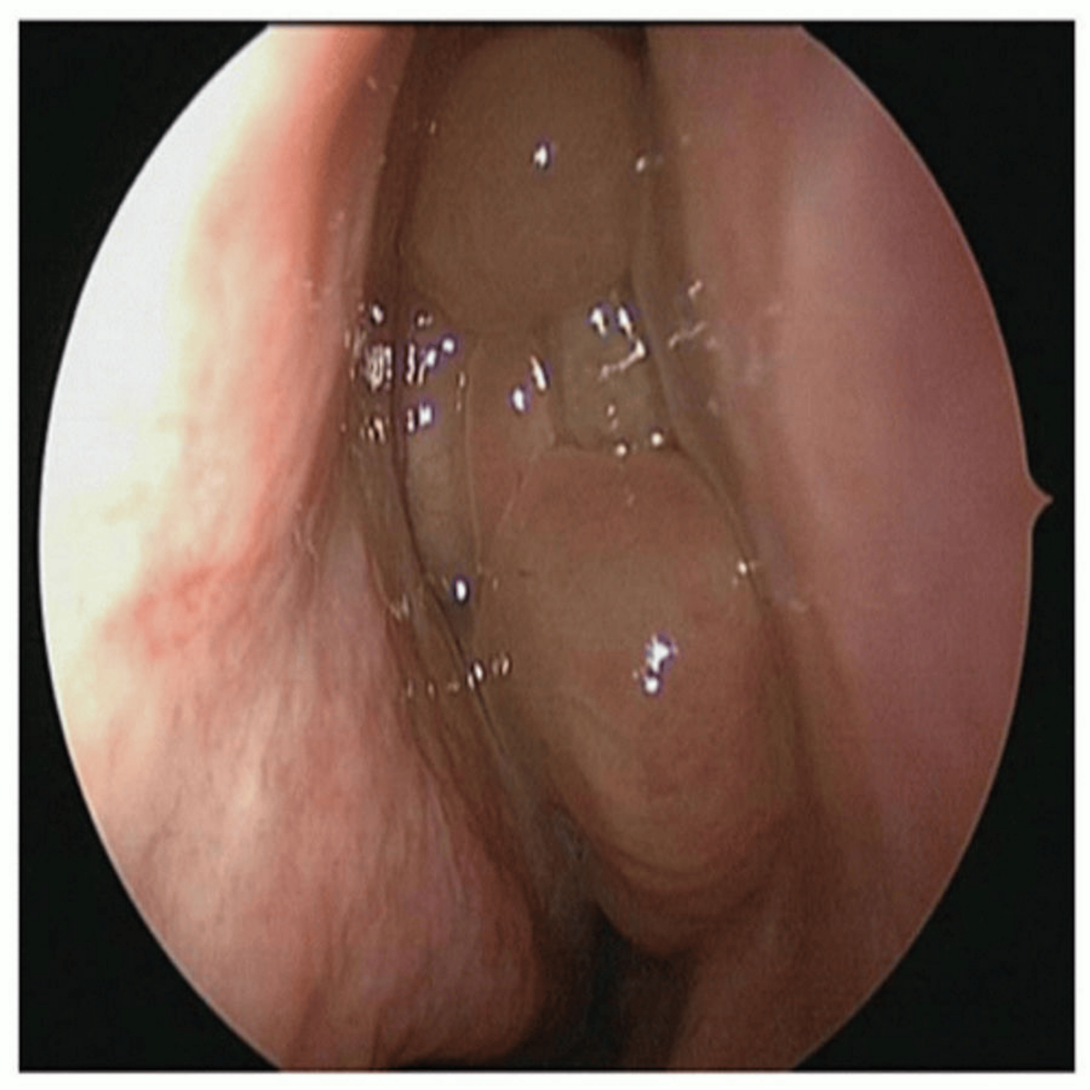
Nasal endoscopy image of case 1. Nasal endoscopy demonstrated mucopurulent discharge emerging from the right sphenoethmoidal recess.

**Figure 2:**
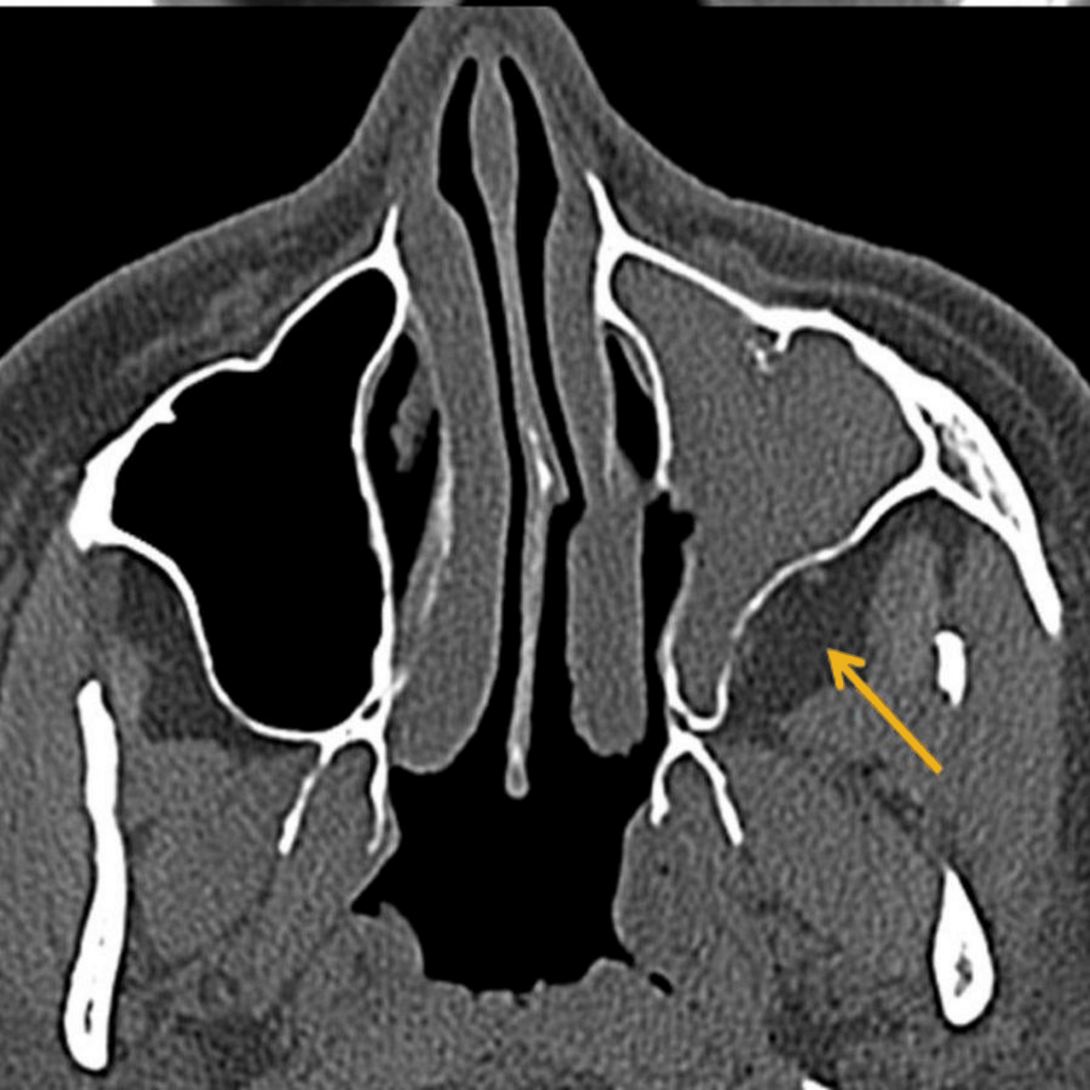
CT paranasal sinus of case 1. CT PNS (yellow arrow) showed isolated soft-tissue opacification of the right sphenoid sinus, without evidence of bony erosion, intracranial extension, or orbital invasion. PNS: paranasal sinuses.

General and systemic examinations were unremarkable. Ophthalmic assessment revealed mild right-sided proptosis, with visual acuity of 6/12 improving to 6/9 with pinhole. Extraocular movements were preserved, and no cranial nerve deficits were elicited. Diagnostic nasal endoscopy demonstrated mucopurulent discharge emerging from the right sphenoethmoidal recess, prompting further evaluation (Figure 1). CT of the paranasal sinuses revealed isolated soft-tissue opacification of the right sphenoid sinus without bony erosion or orbital/intracranial involvement (Figure 2).

She underwent endoscopic sphenoidotomy under general anesthesia. Thick necrotic material was evacuated, and histopathology demonstrated broad, ribbon-like, pauciseptate hyphae, confirming mucormycosis. Postoperatively, she was initiated on intravenous liposomal amphotericin B. Over subsequent follow-up visits, her visual acuity gradually improved to 6/6, and the proptosis resolved completely, consistent with recovery following timely sphenoid decompression (Table 2).

Case 2

A 52-year-old woman with a 12-year history of poorly controlled diabetes, along with hypertension and bronchial asthma, presented with an acute one-week history of right periorbital swelling, progressive blurring of vision, and horizontal diplopia. She reported a dull aching headache but no nasal symptoms or preceding respiratory infection.

**Figure 3:**
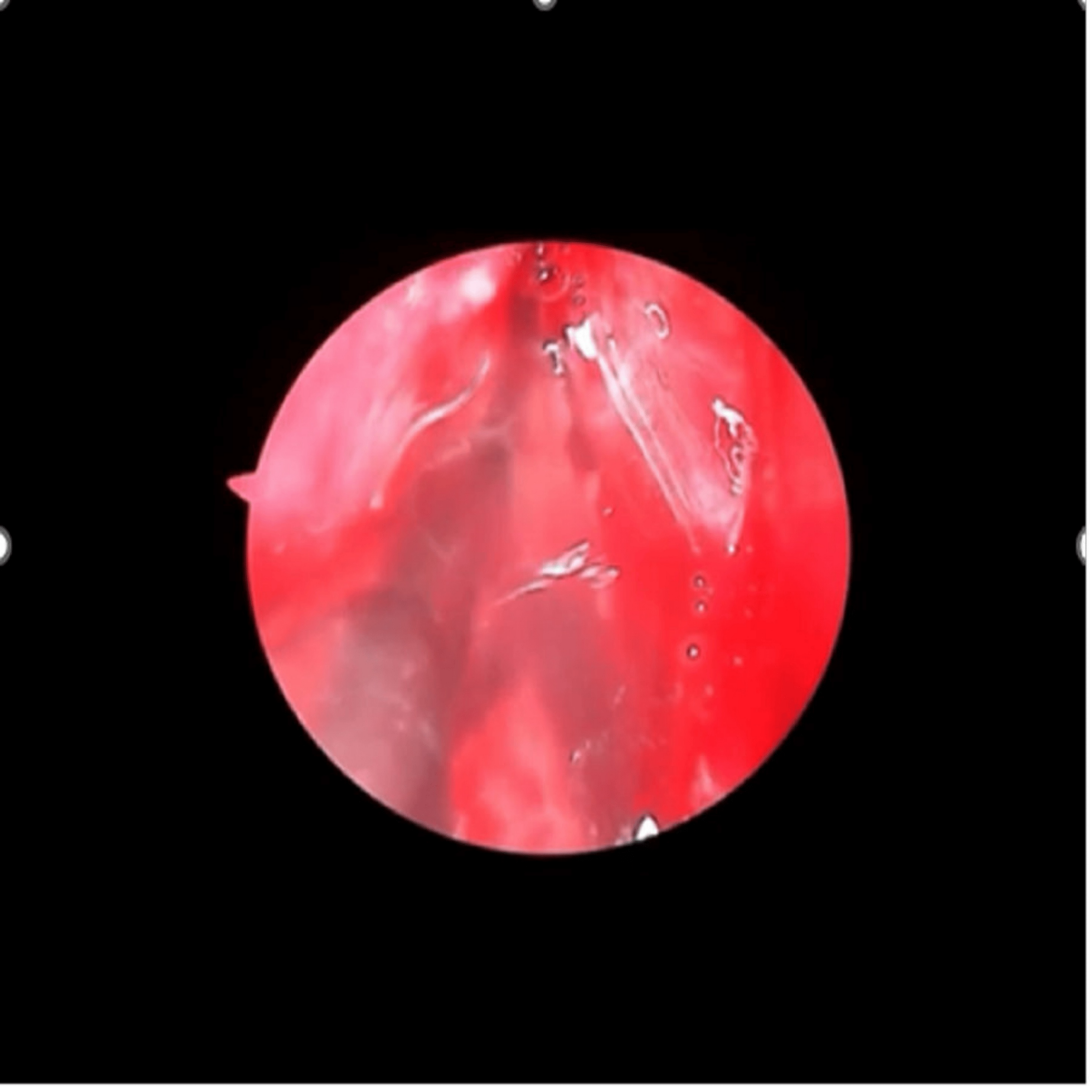
Intraoperative nasal endoscopy of case 2. Intraoperative nasal endoscopy of the right sphenoid sinus showing fungal debris while giving a wash to clear the sinus.

**Figure 4:**
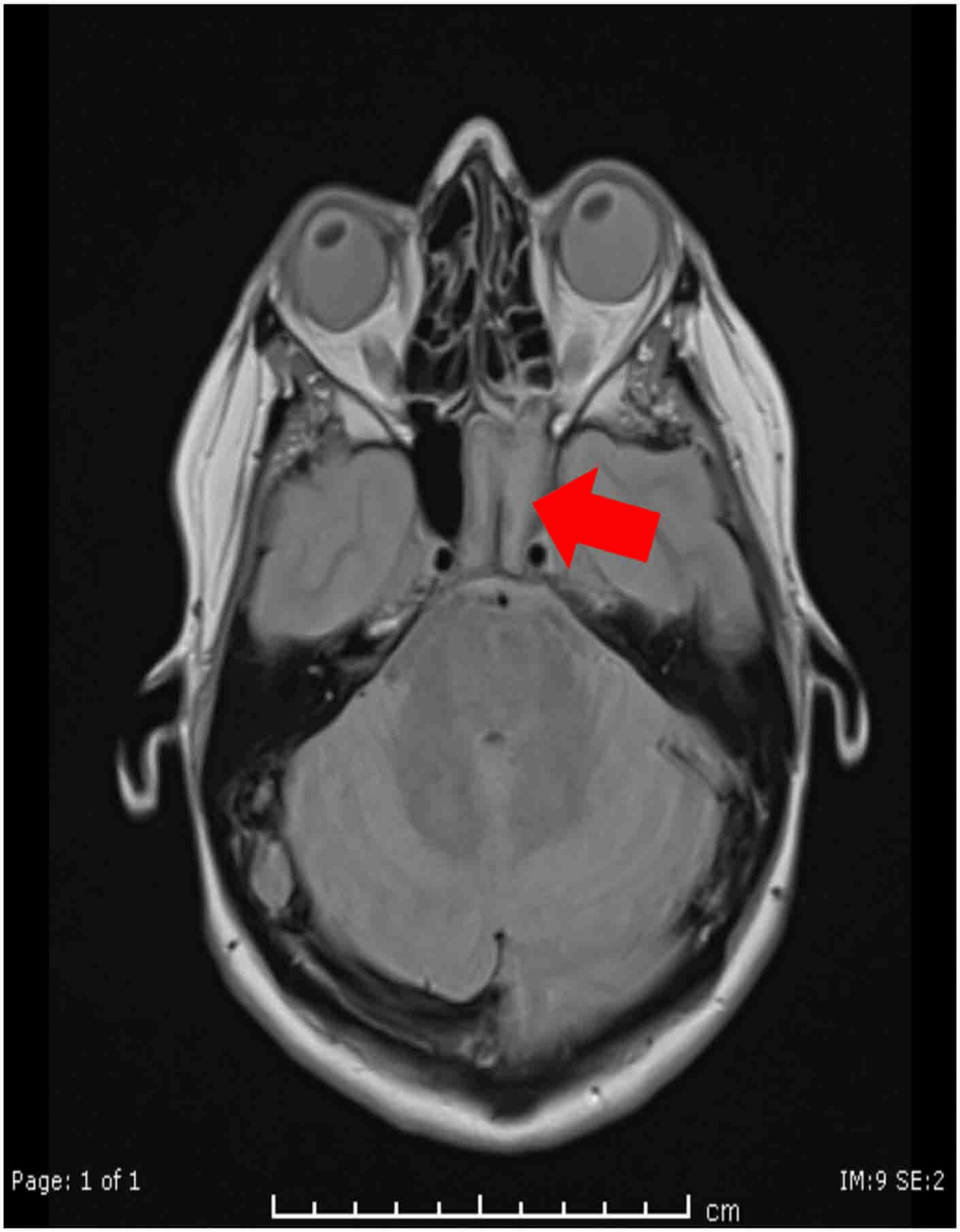
CT of paranasal sinuses of case 2. Computed tomography of the paranasal sinuses demonstrated soft tissue opacification (red arrow) in the sphenoid sinus. IM: inferior meatus; SE: sphenoethmoidal recess.

**Figure 5:**
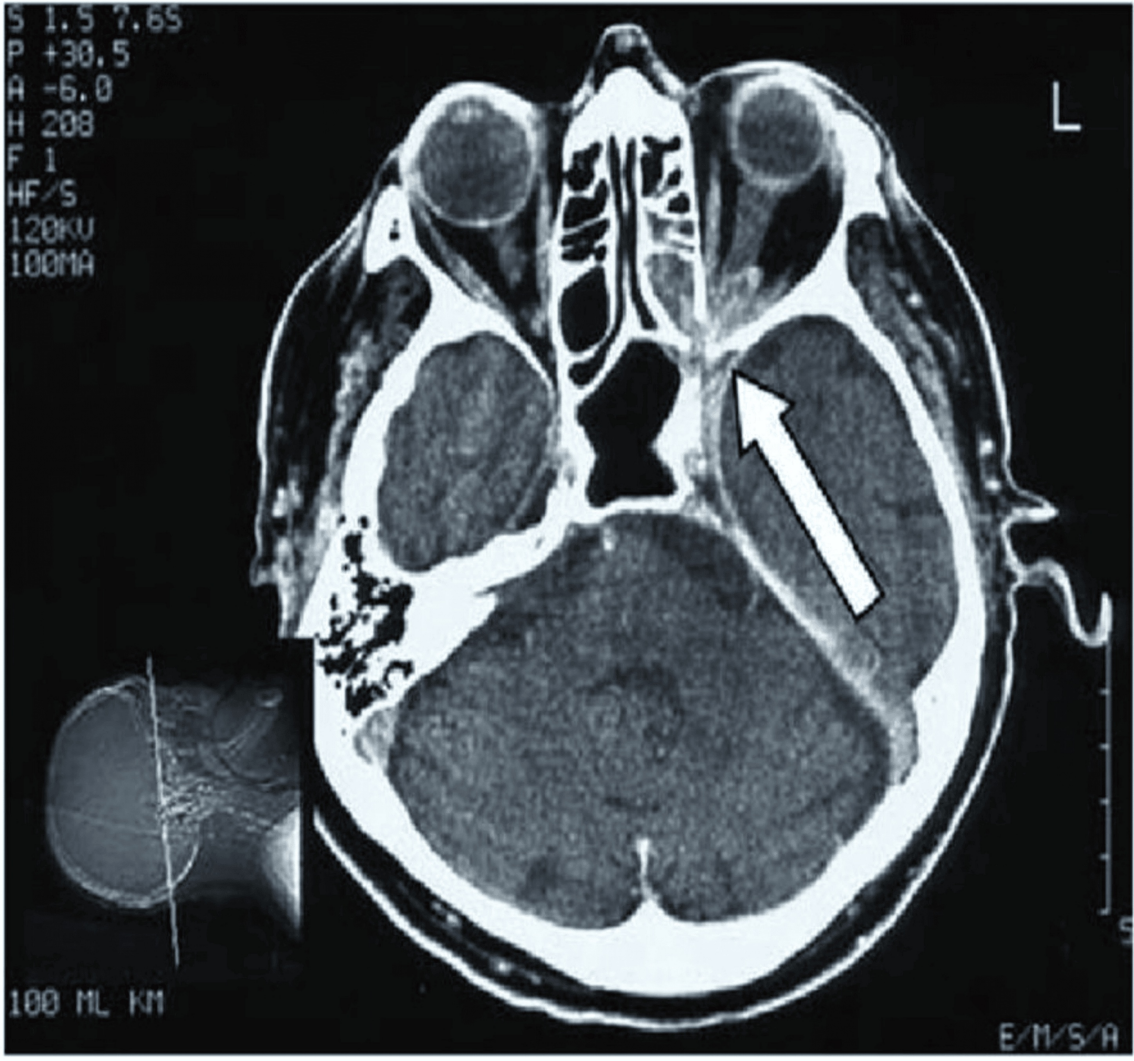
MRI brain with contrast of case 2. MRI with contrast demonstrated soft tissue opacification (arrow) in the sphenoid sinus with extension toward the orbital apex.

Clinical examination revealed right eye visual acuity of 6/18, improving to 6/9, with evident proptosis, ptosis, and restriction of abduction. The pattern suggested involvement of cranial nerves III, IV, and VI, indicative of an emerging orbital apex syndrome. Diagnostic nasal endoscopy showed right-sided septal deviation with mucosal edema near the sphenoethmoidal region (Figure 3). CT PNS and contrast-enhanced MRI revealed sphenoid sinus opacification with extension toward the orbital apex, without frank bony erosion or cavernous sinus thrombosis (Figures 4, 5).

Functional endoscopic sinus surgery was performed, including posterior ethmoidectomy and sphenoidotomy. Inspissated secretions were removed and submitted for laboratory evaluation. Postoperatively, progressive resolution of proptosis and ptosis was observed, followed by restoration of full extraocular movements. Diplopia resolved completely, and visual acuity improved to 6/6, indicating successful decompression of the optic-orbital pathway (Table 2).

Case 3

A 50-year-old woman with a 25-year history of poorly controlled type 2 diabetes mellitus presented with a two-week history of progressive diplopia and blurred vision in the right eye. She denied nasal, ocular, or systemic symptoms, contributing to the delayed suspicion of sphenoid sinus pathology.

**Figure 6:**
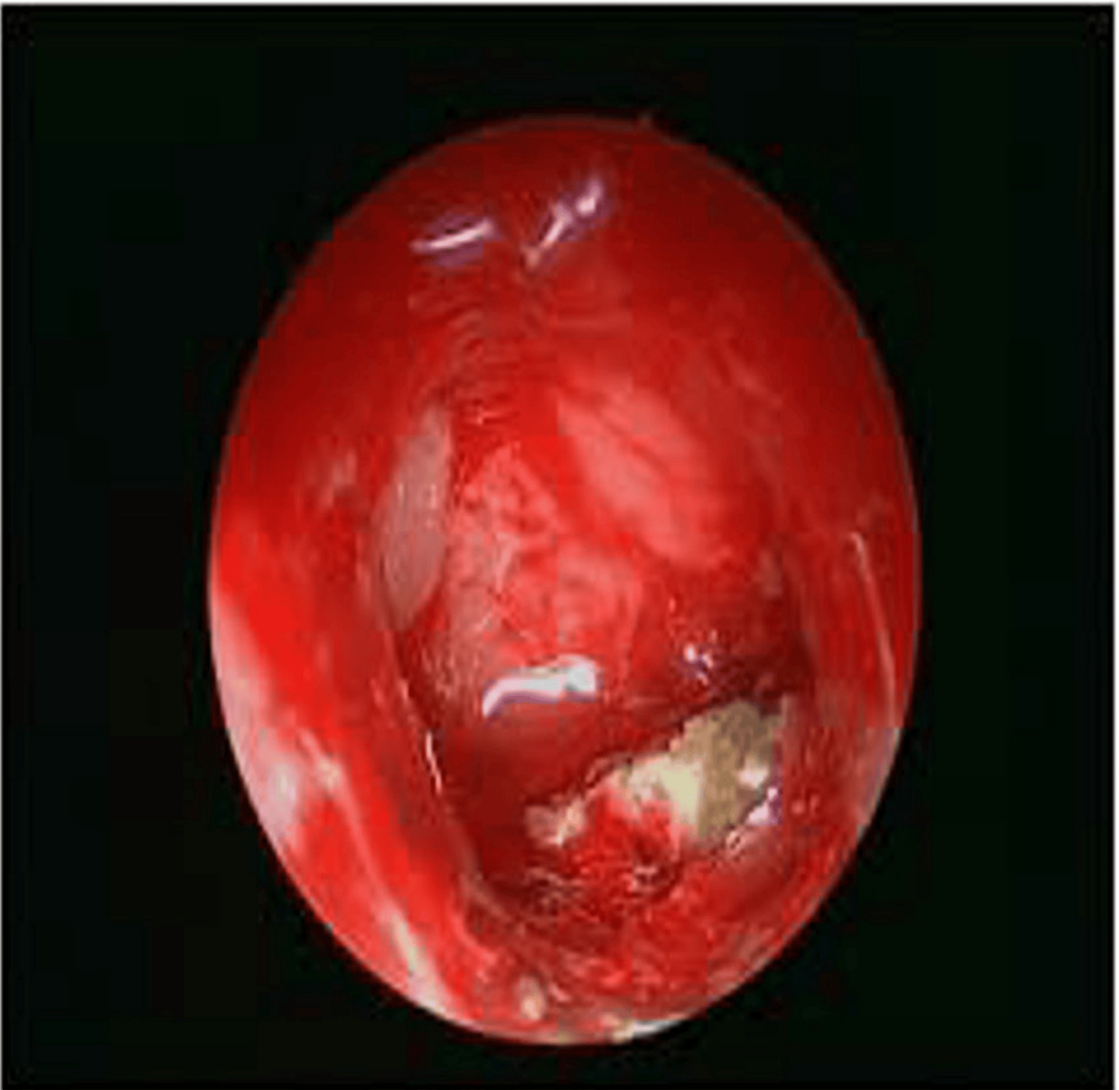
Intraoperative nasal endoscopy of case 3. Intraoperative nasal endoscopy of the right sphenoid sinus showing fungal debris on the floor of the sphenoid sinus.

**Figure 7:**
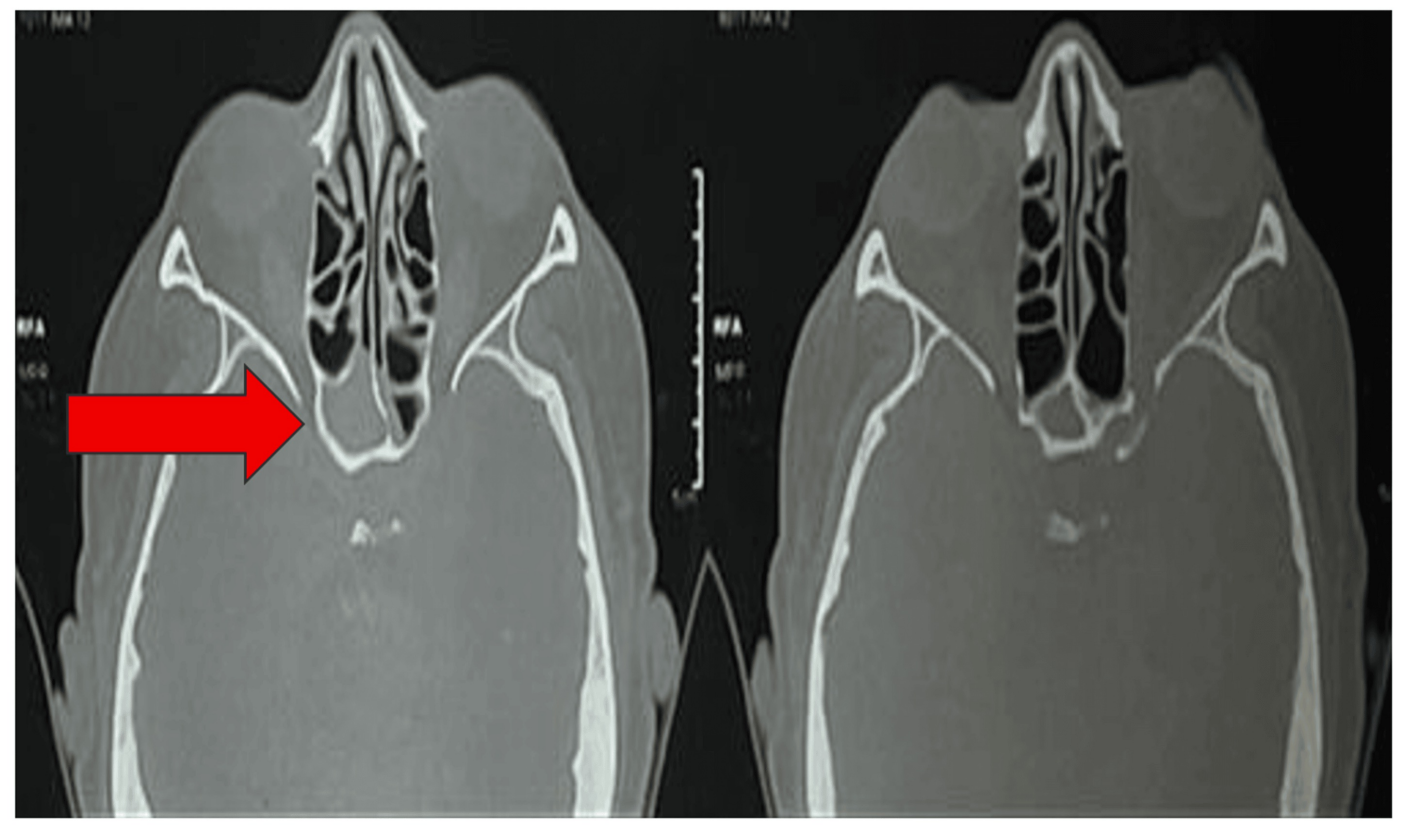
CT paranasal sinus of case 3. CT imaging demonstrated isolated mucosal disease (red arrow) in the right sphenoid sinus.

Examination showed stable vital parameters and right eye visual acuity of 6/18 improving to 6/9. Lateral gaze diplopia corresponded to an isolated right abducens (cranial nerve VI) palsy. Diagnostic nasal endoscopy revealed minimal mucosal congestion without discharge (Figure 6). CT imaging showed isolated right sphenoid mucosal disease without orbital or intracranial extension (Figure 7).

She underwent endoscopic sphenoidotomy, and thick fungal debris was drained. Mycological analysis demonstrated septate hyphae with acute-angle branching, consistent with Aspergillus species, indicative of chronic invasive fungal sinusitis or a fungal ball. Postoperatively, her lateral gaze limitation resolved gradually, and diplopia disappeared entirely. Visual acuity returned to 6/6, with no recurrence noted on follow-up nasal endoscopy (Table 2).

## Discussion

In this case series, three female patients between 50 and 53 years of age, all with poorly controlled type 2 diabetes mellitus, presented predominantly with visual disturbances rather than sinonasal symptoms, highlighting the insidious nature of sphenoid sinus pathology. Each patient exhibited distinct ophthalmic manifestations: gradual visual blurring with mild proptosis in case 1, acute orbital swelling with ptosis and extraocular muscle restriction indicative of orbital apex syndrome in case 2, and isolated abducens nerve palsy with diplopia in case 3. Across all cases, nasal findings were minimal on anterior rhinoscopy and diagnostic nasal endoscopy, while imaging consistently localized disease to the sphenoid sinus. This pattern underscores the characteristic tendency of posterior sinus disease to remain clinically silent until orbital or neurological structures become compromised.

The clinical profiles observed align closely with established descriptions of posterior paranasal sinus mycoses. Chakrabarti et al. reported that diabetic patients are particularly susceptible to sphenoid sinus fungal infections and frequently present with cranial neuropathies or visual impairment due to the sinus's close anatomical relationship with the cavernous sinus and orbital apex [10]. Similarly, in our series, ophthalmic symptoms were the initial presenting feature, leading to early referral through ophthalmology rather than otolaryngology pathways.

All three patients underwent endoscopic sphenoidotomy via the transnasal approach, which offers direct access to the sphenoethmoidal recess. Stammberger et al. emphasized the safety and efficacy of this technique in achieving adequate ventilation, drainage, and debridement of the sphenoid sinus while minimizing morbidity [11]. The uniformly favorable postoperative outcomes in the present series further reinforce the central role of timely endoscopic intervention in preventing irreversible neuro-ophthalmic damage.

Case 1 demonstrated mucormycosis, a well-known angioinvasive fungal infection. Walsh et al. emphasized that mucormycosis demands early clinical suspicion and urgent intervention due to its potential to cause ischemic neuropathy, vascular thrombosis, and rapid intracranial extension [12]. Spellberg et al. similarly highlighted angioinvasion as a key pathogenic mechanism underlying visual loss and cranial nerve dysfunction in mucormycosis [13]. In concordance with these observations, the complete visual recovery to 6/6 following prompt surgical decompression and initiation of intravenous liposomal Amphotericin B illustrates the significance of early recognition and aggressive management.

Case 2 presented with orbital apex syndrome, characterized by involvement of cranial nerves III, IV, and VI, along with proptosis and ptosis. Honavar et al. proposed a staging algorithm for rhino-orbito-cerebral mucormycosis, stressing that orbital apex involvement requires immediate surgical and medical therapy to prevent permanent visual deficits [14]. While no bony erosion was evident radiologically, Hosseini et al. documented that fungal pathogens may extend to the orbital apex through thin bony partitions or via venous channels, even in the absence of overt destructive changes [15]. MRI was instrumental in delineating soft-tissue involvement in this patient, consistent with the findings of Groppo et al., who demonstrated MRI’s superior sensitivity in detecting early orbital and perineural infiltration compared with CT imaging [16]. Following timely functional endoscopic sinus surgery (FESS) with posterior ethmoidectomy and sphenoidotomy, this patient experienced complete restitution of extraocular movements and visual acuity, affirming the reversibility of compressive neuropathy when addressed promptly.

Case 3 exhibited isolated abducens nerve palsy due to chronic invasive Aspergillus sphenoid sinus disease. Chakrabarti et al. described chronic Aspergillus sinusitis in diabetics as a subtle, slowly progressive condition often lacking overt sinonasal symptoms, a pattern mirrored in this patient’s presentation [17]. Lin et al. specifically identified cranial nerve VI palsy as a hallmark of sphenoid Aspergillus involvement due to the nerve’s anatomical trajectory along the lateral cavernous sinus [18]. Histopathological confirmation of septate, acute-angle branching hyphae was consistent with Aspergillus morphology described by Kousha et al. [19]. As in the preceding cases, endoscopic sphenoidotomy resulted in full symptomatic resolution and restoration of visual acuity.

The uniformly positive outcomes observed across all three cases echo the conclusions of Turner et al., who demonstrated that early endoscopic debridement significantly improves functional and visual outcomes in invasive fungal sinus disease [9]. Additionally, Soler and Smith highlighted that restoration of sinus aeration via FESS promotes improved mucociliary function and reduces long-term recurrence risk, supporting the sustained postoperative recovery documented in this series [20].

## Conclusions

This case series highlights the deceptive nature of isolated sphenoid sinus disease, particularly in diabetic individuals, where ophthalmic symptoms may precede or overshadow sinonasal complaints, leading to delayed diagnosis. The three cases demonstrate that visual disturbances, cranial neuropathies, and orbital apex involvement can occur even in the absence of significant nasal findings, underscoring the need for heightened clinical suspicion in high-risk patients. Early imaging, prompt endoscopic sphenoidotomy, and appropriate antifungal therapy, especially in mucormycosis and Aspergillus-related disease, proved crucial in achieving complete visual and neurological recovery. These findings reaffirm that timely multidisciplinary intervention can significantly reduce morbidity and prevent irreversible vision loss in patients with posterior sinus pathology.
